# Knowledge, attitudes, and practices of health providers regarding access to and use of contraceptive methods among adolescents and youth in urban Guinea

**DOI:** 10.3389/fpubh.2022.953806

**Published:** 2022-11-17

**Authors:** Sidikiba Sidibé, Delphin Kolié, Fassou Mathias Grovogui, Karifa Kourouma, Bienvenu Salim Camara, Alexandre Delamou, Seni Kouanda

**Affiliations:** ^1^Institut Africain de Santé Publique (IASP/USTA) of the University Saint Thomas D'Aquin, Ouagadougou, Burkina Faso; ^2^African Centre of Excellence in the Prevention and Control of Communicable Diseases (CEA-PCMT), Faculty of Sciences and Health Techniques, Gamal Abdel Nasser University of Conakry, Conakry, Conakry, Guinea; ^3^National Training and Research Centre in Rural Health of Maferinyah, Forecariah, Guinea

**Keywords:** contraception, knowledge, attitudes and practices, health provider, adolescents and youth, Guinea

## Abstract

**Introduction:**

The objective of this study was to analyze providers' knowledge, attitudes, and practices regarding access to and use of contraception by urban adolescents and youth.

**Methods:**

This is a cross-sectional study of 1,707 health care providers in 173 selected private and public health facilities in the capital city of Conakry and the seven administrative regions of Guinea. Factors associated with health care providers' attitudes and practices were then analyzed using logistic regression.

**Results:**

Among the 1,707 health providers, 71% had a good level of Knowledge about modern contraceptive use among adolescents and youth. In addition, 62% had positive attitudes, and 41% had good prescribing practices toward using modern contraceptive methods by adolescents and youth. Being a midwife (aOR: 1.39, 95%CI: 1.02–1.89), Being aged 25–34 years (aOR: 1.7, 95%CI: 1.2–2.3), 35–44 years (aOR: 2.1, 95%CI: 1.4–3.0), and 45 years, and older (aOR: 2.4, 95%CI: 1.3–4.2), an increase of years in professional experience (aOR:1.05; 95%CI: 1.02–1.08) were factors significantly associated with provider positive attitudes. However, being a medical doctor (aOR: 2.37, 95%CI: 1.04–4.42), an increase of years in professional experience (aOR: 1.07; 95%CI: 1.04–1.10) and a positive attitude (aOR: 3.16. 95%CI: 2.48–4.01) were factors associated with good practice in delivering modern contraceptive methods to adolescents and youth.

**Conclusion:**

Positive attitudes and good practices toward the use of contraceptive services by adolescents and youth were found among providers. However, many health care providers still have unfavorable attitudes and practices toward delivering FP services to urban adolescents and youth. Therefore, future intervention programs should focus on training health care providers in youth- and adolescent-friendly reproductive health services and promoting contraception among adolescents.

## Introduction

Despite the relative decline in the overall adolescent birth rate worldwide, from 56 births per 1,000 women aged 15–19 in 2000 to 44 per 1,000 in 2018, it remains high (101 per 1,000) in sub-Saharan Africa (1).

Thus, reducing the frequency of pregnancies among adolescents and young people is becoming a priority in sub-Saharan Africa, where frequent complications related to unwanted pregnancies (2, 3). In this context, although the provision of family planning (FP) services is guided by the principle of informed client choice, a number of barriers limit their availability and use (4, 5). These barriers partially sustained high unmet needs, particularly among adolescents and youth in sub-Saharan Africa contexts (4–6).

In Guinea, half the population is under 24 years. However, in 2018, more than a third (38%) of sexually active adolescent and young unmarried women aged 15–24 had an unmet need for FP (7). The existence of the unmet need for FP can result from factors including lack of delivery of services (e.g., low coverage or quality of FP services, attitudes, and practices of providers) or demand (Knowledge of FP, socio-cultural norms, access to services, etc.) (8, 9). For example, certain socio-cultural norms or individual considerations may influence health care providers' attitudes that limit adolescents' and youths' access to and use of sexual and reproductive health services (10). Thus, provider attitudes and communication problems, real or perceived, may contribute to the unmet needs for contraception (2, 11).

In addition, several studies in Africa have found that providers have restrictive practices or attitudes toward the delivery of contraceptive services to adolescents and youth (12–15). In this context, non-marital sexual relations are very poorly perceived by some health providers, leading to negative attitudes on their part in providing FP services to unmarried young women (12, 16). Other providers reported feeling very uncomfortable or reluctant about delivering a modern contraceptive method to adolescents and youth because of their young age (6, 17). In Senegal, a study examined the role of provider restrictions on young women's access to and use of contraception. These biases and provider restrictions contributed to youth's lack of access to contraception (18).

Furthermore, the perception that contraceptive use can lead to infertility or that it represents a practice contrary to religious belief justifies the negative attitude of some providers (19, 20). Moreover, providers' lack of knowledge about the eligibility criteria for modern contraceptive methods among adolescents and youth encourages irregular prescription of contraceptives (21).

However, favorable attitudes toward contraception among youth were most likely to be found among health providers with more education or those who had received sufficient continuing education on adolescent sexuality and reproductive health (16, 20, 22). Thus, health providers need sufficient knowledge and a positive attitude toward adolescent contraceptive users to better play their role in prescribing contraceptives to prevent unplanned pregnancies among adolescents and young women (21).

Although the various studies mentioned above examine the attitudes and perceptions of providers in delivering family planning services to adolescents and young women, certain aspects remain little studied, for example, the specific factors that influence providers' practice in urban areas.

Systematic analysis of the factors associated with the attitudes and practices of these providers would generate useful information for improving contraceptive use in this target population. In addition, it would help to address the challenges that are slowing down the repositioning of FP and the promotion of sexual and reproductive health among adolescents and youth in urban areas (23, 24).

The objective of this study was to analyze providers' knowledge, attitudes, and practices (KAP) regarding access to and use of contraceptive methods by adolescents and youth in urban areas. Specifically, it describes the KAPs and analyzes the factors associated with providers' attitudes and practices.

## Materials and methods

### Type and period of study

This cross-sectional study was conducted between November 1st, 2020, and January 31st, 2021.

### Study setting

The study was conducted in public and private health facilities (hospitals, communal medical centers (CMCs), health centers, and private clinics) in the capitals of the eight (8) administrative regions of Guinea, including the capital, Conakry.

### Study population

The study population consisted of all health care providers (medical doctors, nurses, midwives, and pharmacists) involved in delivering FP services in the study sites' public and private health facilities.

### Sampling

A multistage sampling technique was used to select health facilities and study participants. Then, at the level of each region, we proceeded to the exhaustive selection of national and regional hospitals and CMCs. Then, health centers, private clinics, practices, and NGOs were selected using a simple random sampling procedure according to public or private type.

The list of public and private health facilities or approved associations involved in delivering family planning services was used to select the health facilities participating in the study.

Of the 328 operational health facilities identified, 173 (52.8%), including 19 hospitals/CMCs, 69 health centers, 41 private hospitals/clinics, and 54 private practices or health centers, were surveyed in the five communes of Conakry and 11 health districts. In each of the involved health facilities included in the study, the health providers present in the services on the day of the survey were interviewed.

### Data collection

We used the ODK (Open Data Kit) electronic data collection system to collect data from health workers. A standardized electronic questionnaire, pre-tested and integrated with tablets, was also administered individually to health care workers by previously trained collection agents. Data collection was done under the direct supervision of the principal investigator and his two research assistants.

The information collected focused on the socio-demographic characteristics, knowledge, attitudes, and practices of health workers regarding the use of modern contraceptive methods among adolescents and youth in urban Guinea.

### Variables

#### Outcome variables

The variables of interest in this study were positive attitudes and good practices of health providers toward contraceptive use by adolescents and youth. The design questions for defining the variables were derived from the literature review of studies conducted in Nigeria, Botswana, and Ethiopia (12, 21, 25).

Providers' positive attitudes were defined based on 10 questions on providers' attitudes toward contraceptive use by adolescents and youth. Measurement was done by Likert scales of 0 to 4 points with 0 corresponding to strongly disagree and 4 to strongly agree (26). Providers who scored at or above the mean of 26 out of 40 were considered to have a positive attitude toward contraception for adolescents and youth. Those with a score below the average were considered to have a negative attitude.

Providers' good practices were defined based on three questions for which a Likert scale ranging from 0 (never) to 4 (always) was also used. Providers, who gave positive answers to all the questions, i.e., often, or always prescribe and counsel methods to adolescents and youth and never scold them when they expressed the need to use a contraceptive method, were classified as having good practices. Conversely, those who did not give positive answers to all three questions were classified as not having good practices.

#### Independent variables

The independent variables included the socio-demographic characteristics of providers of FP methods to adolescents and youth and their level of knowledge about FP. Socio-demographic characteristics included age (20–24, 25–34, 35–44, or 45 and over), sex (male or female), marital status (married/married or single/divorced/widowed), socio-professional category (medical doctor, midwife, nurse, nurse-assistant, or pharmacist), religion (Muslim or Christian), number of years of experience, ethnic group (Soussou, Peulh, Malinke, or Forestier), use of modern contraceptives, direct involvement in the provision of FP services, type of facility (public or private), and administrative region (Conakry, Kindia, Boké, Mamou, Labe, Kankan, Faranah, or Nzerekore), and training on FP in the past 12 months.

Knowledge variables (defined as a good level of Knowledge about modern contraceptive use among adolescents and youth) included having high confidence in prescribing contraceptive methods to adolescents and youth; high confidence in explaining contraceptive methods to adolescents and youth; Knowledge of the existence of FP policy and legislation for adolescents and youth, and Knowledge of the rights of adolescents and youth to receive FP before marriage). Confidence variables were measured using a scale ranging from 1 (very poor) to 5 (very good). Those who had four or five scores were classified considering as having high confidence toward prescribing or explaining contraceptive methods to adolescents and youth.

### Data analysis

Descriptive variables were presented as means (with standard deviations) and proportions (with 95% confidence intervals). Knowledge, attitudes, and practices of health care workers were described as proportions. Factors associated with health care workers' attitudes and practices were then analyzed using logistic regression. Multi-collinearity was checked before including the independent variables in the regression model.

Unadjusted and adjusted odds ratios were then calculated with 95% confidence intervals. The significance level was set at 5%. Data were analyzed using Stata 16.0 software (StataCorp 4905 Lakeway Drive College Station, Texas 77845 USA).

## Results

### Socio-demographic characteristics of participants

Table 1 presents the socio-demographic characteristics of providers surveyed about contraceptive use among adolescents and youth.

**Table 1:** Demographic characteristics of surveyed providers on contraception use among adolescents and youth, Guinea, January–March 2021 (*n* = 1,707).

**Variables**	**Number**	**%**
Average age (ET)	32 ± 8.4	
**Age group**		
20–24 years	252	14.8
25–34 years	928	54.4
35–44 years	369	21.6
45 years and more	158	9.3
**Gender**		
Female	1,406	82.4
Male	301	17.6
**Professional category (IQ)**		
Medical doctor	213	12.5
Midwife	799	46.8
Nurse	287	16.8
ATS	361	21.1
Pharmacist	47	2.8
Work experience in FP	4.1 ± 1.2	
**Marital status**		
Married/in union	1,199	70.2
Single/divorced/widowed	508	29.8
**Religion**		
Muslim	1,205	70.6
Christian	502	29.4
**Ethnic group**		
Malinke	515	30.2
Peulh	458	26.8
Forestier	553	32.4
Soussou	181	10.6
**Ever use of FP by the provider**		
No	537	31.5
Yes	1,170	68.5
**FP training in the last 12 months**		
No	1,008	59.1
Yes	699	40.9
**Type of facility**		
Public	1,421	83.2
Private	286	16.8
**Nature of facility**		
Hospital/CMC	457	26.8
HC/HP	957	56.1
Clinic	293	17.2
**Natural region**		
Conakry	450	26.4
Lower Guinea	257	15.1
Upper Guinea	517	30.3
Middle Guinea	195	11.4
Forest Guinea	288	16.9

One thousand seven hundred seven (*n* = 1,707) health care providers were interviewed. One-quarter of them resided in Conakry (*n* = 450; 26.4%), and most were female (*n* = 1,825; 77.7%) and worked in public health facilities (*n* = 1,406; 82.4%). The mean age of the providers was 32 ± 8.4. Midwives were the most represented socio-professional category in the sample (*n* = 799; 46.8%).

In addition, most participants were married or in union (*n* = 1,199; 70.2%) and Muslim (*n* = 1,205; 70.6%), the majority of providers interviewed (*n* = 1,170; 68.5%) had previously used a modern contraceptive method and were directly involved in providing contraceptive services in public health facilities (*n* = 1,421; 83.2%).

### Providers' self-reported confidence in providing FP to youth and awareness of the policy framework

Table 2 shows providers' self-reported confidence in providing FP to youth and knowledge of relevant policies. Most providers reported being confident in explaining the mode, duration of action, advantages, and disadvantages of contraceptive methods (*n* = 1,428; 83.7%) and in prescribing a contraceptive method (*n* = 1,407; 82.4%) to adolescents and youth. The majority of participants reported being aware of the existence of FP legislation policies for adolescents and youth in Guinea (*n* = 991; 58.1%), as well as the right of adolescents and youth aged 15–24 to receive contraception before marriage (*n* = 1,556; 91.2%).

**Table 2:** Providers' knowledge of contraceptive use among adolescents and youth, Guinea, January-March 2021 (*n* = 1,707).

**Variables**	**Number**	**%**
**Confident in explaining FP methods to adolescents/youth**		
Low confidence	279	16.3
High confidence	1,428	83.7
**Confidence in prescribing FP methods to adolescents/youth**		
Low confidence	300	17.6
High confidence	1,407	82.4
**Knowledge of the existence of adolescent FP policy and legislation in Guinea**		
No	716	41.9
Yes	991	58.1
**Knowledge of the right of adolescents/youth (15–24 years) to receive FP before marriage**		
No	151	8.8
Yes	1,556	91.2

### Providers' attitudes about contraceptive use among adolescents and youth

Table 3 displays providers' attitudes about contraceptive use among adolescents and youth. Most providers surveyed agreed that health care staff should provide contraceptive services to married and unmarried adolescents and youth in facilities (*n* = 11,391; 81.5%) and that adolescents and youth should receive contraceptive counseling before becoming sexually active (*n* = 1,519; 89.0%). In addition, more than half of the providers surveyed agreed that unmarried adolescents and youth do not need parental consent before receiving contraceptives (*n* = 937; 54.9%). On the other hand, half of the providers interviewed were opposed stating that adolescents or unmarried youth should not receive contraceptives because their culture does not support premarital sex (*n* = 904 53.0%) and that it is better to tell adolescents and youth to abstain from sex when they ask for contraceptives rather than provide them (*n* = 1,030; 60.3%). Furthermore, two-thirds of participants felt that it is not immoral for adolescents and youth to use contraceptives (*n* = 1,146; 67.1%) and that their religion allows their use (*n* = 835; 48.9%). Overall, about six out of ten providers (61.6%) had positive attitudes toward the use of modern contraceptive methods by adolescents and youth.

**Table 3:** Providers' attitudes about contraceptive use among adolescents and youth, Guinea, January-March 2021 (*n* = 1,707).

**Variables**	**Disagreeing (%)**	**Neutral (%)**	**Agreeing (%)**
I am comfortable with prescribing contraceptives for adolescents and youth	121 (7.1)	57 (3.3)	1,529 (89.6)
Health personnel should provide contraceptive services to married and unmarried adolescents and youth in facilities	252 (14.8)	64 (3.7)	1,391 (81.5)
Adolescents and youth should receive counseling on contraception before becoming sexually active	160 (9.4)	28 (1.6)	1,519 (89.0)
Unmarried adolescents and youth should not have parental consent before receiving contraceptives	633 (37.1)	137 (8.0)	937 (54.9)
My religious belief influences the way I prescribe contraceptives	898 (52.6)	149 (8.7)	660 (38.7)
Giving contraceptives to teens or unmarried youth can promote sexuality	516 (30.2)	138 (8.1)	1,053 (61.7)
Teenagers or unmarried youth should not be given contraceptives because our culture does not support premarital sexual intercourse	904 (53.0)	139 (8.1)	664 (38.9)
It is better to tell adolescents and youth to abstain from sexual intercourse when they ask for contraceptives than giving them contraceptives	1,030 (60.3)	117 (6.9)	560 (32.8)
My religion does not allow the use of contraception by unmarried adolescents and youth	835 (48.9)	148 (8.7)	724 (42.4)
It is immoral for adolescents and youth to use contraceptives	1,146 (67.1)	132 (7.7)	429 (25.1)

### Provider practices regarding contraceptive use among adolescents and youth

Table 4 presents providers' practices regarding FP service use by adolescents and youth. Providers who prescribed contraceptives to adolescents and youth predominated in our study (*n* = 890; 52.1%). Most of them reported that they counsel advise adolescents and youth on the use of contraceptives (*n* = 1,185; 69.4%) and that they never scold adolescents and youth when they ask for contraceptives (*n* = 1,368; 80.1%). Providers who gave positive responses to all three questions were classified as having “good” practices regarding contraceptive provision to adolescents and youth. Overall, two in five (41.4%) were thus classified.

**Table 4:** Provider practices regarding contraceptive use among adolescents and youth, Guinea, January-March 2021 (*n* = 1,707).

**Variables**	**Never**	**Rarely**	**Always**
Prescription contraceptives to adolescent and youth	139 (8.1)	678 (39.7)	890 (52.1)
Counseling adolescents and youth on the use of contraceptives	105 (6.2)	417 (24.4)	1,185 (69.4)
Scolding adolescents and youth when they ask for contraceptives	1,368 (80.1)	209 (12.2)	130 (7.6)

### Factors associated with providers' attitudes and practices regarding contraceptive use among adolescents and youth

Table 5 presents factors associated with providers' attitudes about contraceptive use among adolescents and youth.

**Table 5:** Factors associated with provider attitudes toward contraceptive use among adolescents and youth, Guinea, January-March 2021 (*N* = 1,707).

**Variables**	**cOR**	**IC95%**		***p*–value**	**aOR**	**CI95%**		***p*–value**
		**Lower**	**Upper**			**Lower**	**Upper**	
**Age group**								
20–24 years	Ref.				Ref.			
25–34 years	1.77	1.34	2.35	<0.001	1.68	1.24	2.26	0.001
35–44 years	2.49	1.79	3.46	<0.001	2.05	1.41	2.98	<0.001
45 years and more	3.29	2.13	5.08	<0.001	2.35	1.32	4.17	0.004
**Gender**								
Female	1.14	0.89	1.47	0.309				
Male	Ref.							
**Professional categories**								
Medical doctor	0.97	0.68	1.38	0.857	1.15	0.77	1.73	0.502
Midwife	1.34	1.02	1.76	0.038	1.39	1.02	1.89	0.038
Nurse	Ref.				Ref.			
ATS	1.26	0.92	1.72	0.159	0.95	0.67	1.34	0.765
Pharmacist	0.93	0.50	1.73	0.815	0.95	0.49	1.86	0.890
**Experience (Average)**	1.07	1.04	1.09	<0.001	1.050	1.020	1.080	0.003
**Marital status**								
Married/in union	1.12	0.91	1.39	0.283				
Single/divorced/widowed	Ref.							
**Religion**								
Muslim	Ref.				Ref.			
Christian	2.15	1.71	2.70	<0.001	1.18	0.71	1.96	0.521
**Ethnic groups**								
Malinke	1.28	0.99	1.65	0.057	0.98	0.74	1.32	0.912
Peulh	Ref.				Ref.			
Forestier	2.58	1.98	3.35	<0.001	1.69	1.01	2.85	0.047
Soussou	1.25	0.88	1.77	0.210	1.31	0.90	1.90	0.688
**Ever use of FP by the provider**								
No	Ref							
Yes	0.99	0.80	1.22	0.888				
**FP training in the last 12 months**								
No	Ref							
Yes	1.10	0.83	1.23	0.931				
**Type of facility**								
Public	1.50	1.16	1.94	0.002	0.94	0.70	1.26	0.688
Private	Ref.				Ref.			
**Natural region**								
Conakry	Ref.				Ref.			
Lower Guinea	1.53	1.12	2.09	0.007	1.48	1.05	2.07	0.023
Upper Guinea	2.15	1.66	2.80	<0.001	2.67	1.92	3.71	<0.001
Middle Guinea	1.13	0.81	1.58	0.478	1.47	1.01	2.15	0.047
Forest Guinea	2.92	2.11	4.03	<0.001	2.73	1.84	4.04	<0.001

The bivariate analysis showed statistically significant associations between providers' positive attitude toward the use of modern contraceptive methods by adolescents and youth and the following variables: age, socio-professional category, and experiences in the provision of FP services, religion, ethnic group, and type of facility and region of residence.

However, after adjustment using logistic regression, only age, socio-professional category, number of years of experience in the provision of FP services, ethnic group, and region of residence were statistically significantly associated with providers' positive attitude toward the use of modern contraceptive methods by adolescents and youth. Indeed, compared to young providers (20–24 years), those aged 25–34 years (aOR: 1.68, 95%CI: 1.24–2.26), 35–44 years (aOR: 2.05, 95%CI: 1.41–2.98) and 45 years and older (aOR: 2.35, 95%CI: 1.32–4.17) were respectively 1.7–2.4 times more likely to have a positive attitude toward the use of modern contraceptive methods by adolescents and youth. Midwives (aOR: 1.39, 95%CI: 1.02–1.89) were 1.39 times more likely to have a positive attitude than another professional category. Providers residing in lower Guinea (aOR: 1.48, 95%CI: 1.05–2.07), Middle Guinea (aOR: 1.47, 95%CI: 1.01–2.15), Forest Guinea (aOR: 2.73, 95%CI: 1.84–4.04) and Upper Guinea (aOR: 2.67, 95%CI: 1.92–3.71) had better attitudes toward the use of modern contraceptive methods by adolescents and youth compared to providers in Conakry. Positive provider attitude increased significantly by 5% for each additional year of experience (*p* = 0.003).

The bivariate analysis in Table 6 shows statistically significant associations between provider practices and the following variables: age, gender, socio-professional category, ethnic group, provider use of FP, type of facility, positive attitude of health providers, number of years of experience, and region of residence.

**Table 6:** Factors associated with providers' good practices in prescribing contraceptive methods to adolescents and youth, Guinea, January-March 2021, (*N* = 1,707).

**Variables**	**cOR**	**IC95%**		***p*-value**	**aOR**	**CI95%**		***p*-value**
		**Lower**	**Upper**			**Lower**	**Upper**	
**Age group**								
20–24 years	Ref.				Ref.			
25–34 years	1.63	1.20	2.20	0.002	1.28	0.90	1.82	0.171
35–44 years	2.12	1.51	2.98	<0.001	1.23	0.80	1.89	0.339
45 years and more	3.80	2.50	5.78	<0.001	1.77	0.97	3.24	0.065
**Gender**								
Female	1.34	1.03	1.73	0.027	1.19	0.81	1.75	0.376
Male	Ref.				Ref.			
**Professional categories**								
Medical Doctor	1.86	0.91	3.79	0.087	2.37	1.04	5.42	0.041
Midwife	2.70	1.38	5.28	0.004	2.20	0.99	4.90	0.054
Nurse	1.23	0.61	2.48	0.568	1.23	0.55	2.75	0.623
ATS	1.98	0.99	3.94	0.052	1.63	0.72	3.68	0.239
Pharmacist	Ref.				Ref.			
**Experience**	1.1	1.08	1.12	<0.001	1.07	1.04	1.1	<0.001
**Marital status**								
Mary/in union	1.13	0.91	1.39	0.269				
Single/divorced/widowed	Ref.							
**Religion**								
Muslim	Ref.				Ref.			
Christian	0.87	0.70	1.08	0.204	0.92	0.56	1.52	0.743
**Ethnic group**								
Malinke	170	1.32	2.21	<0.001	1.50	1.09	2.06	0.013
Peulh	Ref.				Ref.			
Forestier	1.27	0.98	1.64	0.069	1.50	0.89	2.55	0.131
Soussou	1.48	1.04	2.09	0.030	1.18	0.78	1.79	0.436
**Use of FP by the provider**								
No	Ref.				Ref.			
Yes	1.53	1.24	1.89	<0.001	1.17	0.90	1.52	0.233
**FP training in the last 12 months**								
No	Ref.				Ref.			
Yes	1.19	0.98	1.44	0.085	1.04	0.83	1.30	0.742
**Type of facility**								
Public	1.56	1.20	2.04	0.001	0.92	0.66	1.28	0.637
Private	Ref.				Ref.			
**Natural region**								
Conakry	Ref				Ref.			
Lower Guinea	4.36	3.14	6.04	<0.001	4.50	3.06	6.62	<0.001
Upper Guinea	2.86	2.19	3.72	<0.001	2.98	2.08	4.27	<0.001
Middle Guinea	0.93	0.64	1.34	0.701	1.25	0.90	1.93	0.327
Forest Guinea	0.66	0.47	0.92	0.017	0.59	0.39	0.91	0.018
**Positive attitude**								
No	Ref.				Ref.			
Yes	3.16	2.55	3.91	<0.001	3.16	2.48	4.01	<0.001

After adjustment, only socio-professional category, ethnic group, number of years of experience in the provision of FP services and region of residence, and providers' positive attitude were significantly associated with good provider practice in offering modern methods to adolescents and youth. Physicians (aOR: 2.37, 95%CI: 1.04–5.42) were twice as likely to have good practice compared to pharmacists. Ethnic group Malinke (aOR: 1.50, 95%CI: 1.09–2.06) were about 1.5 times more likely to have good practices compared to Peulh ethnic group.

Providers residing in lower Guinea (aOR: 4.50, 95%CI: 3.06–6.62), Upper Guinea (aOR: 2.98, 95%CI: 2.08–4.27) were more favorable in offering modern contraceptive methods services to adolescents and youth compared to providers in Conakry. Having good practice to provide modern contraceptive method services was significantly associated with positive provider attitude toward adolescents and young (aOR: 3.16, 95%CI: 2.48–4.01). In addition, favorable practice increased significantly by 7% for each additional year of experience (*p* < 0.001) in providing modern method service delivery to adolescents and young.

## Discussion

This study is one of the first to explore FP providers' knowledge, attitudes, and practices with regard to adolescents and youth and their determinants in Guinea.

The data from the survey of providers' attitudes and practices regarding the provision of FP counseling and services to adolescents and youth revealed a degree of tension between personal moral beliefs and perceived obligation or duty to provide services. Substantial minorities (close to 40%) of providers thought that teenagers and unmarried youth should abstain from sex and that contraceptive use by them was immoral and contrary to religious beliefs and cultural norms. A majority thought that provision of contraception would only encourage sexuality. Yet close to 90% were aware of the right of young people to receive family planning advice and services and over 80% agreed that health personnel should provide contraceptive services to unmarried as well as married young people. Similar high proportions expressed confidence in counseling and service provision to young people and only 15% admitted that they sometimes or often scolded young people when they requested contraceptives.

Interpretation of these findings should be cautious because of the danger that responses may have been influenced by social desirability bias. Yet it does appear probable that most providers with moralistic reservations about premarital sex and contraception do manage to put aside their personal feelings when confronted by young people requesting FP advice and services. This verdict is supported by results from the 2018 DHS in which over 40% of sexually active single women aged 15–24 reported use of injectable or implants (7), both methods that require a visit to a health facility and attention from a trained provider.”

Factors simultaneously associated with positive attitudes and good practices among providers in this study included socio-professional category, number of years of experience in delivering contraceptive services, and region of residence. In addition, provider age was associated with positive provider attitudes in delivering modern methods to adolescents and youth.

About four out of five health care providers were aware of national policies and rights regarding provision of modern contraceptive use among adolescents and youth. The majority (83.7%) reported being confident in explaining the mode, duration of action, advantages, and disadvantages of contraceptive methods to adolescents and youth. Our results are consistent with those of Tshitenge et al. (21) in Botswana, who found that 91.2% of providers felt confident in explaining to adolescents how to use contraceptive methods. There are several possible explanations for this result. In recent years, considerable efforts have been made to improve the availability and use of contraceptive methods by the population, particularly adolescents and youth. For example, the national FP repositioning plan (2014–2018) aimed, among other things, to stimulate the delivery of FP services in all health facilities, including private ones; as well as to strengthen the training program and the equipment of private and public health facilities (27). The effects of these strategies, including the repositioning plan, could explain the level of knowledge of the providers interviewed in our study. In addition, most of the providers interviewed aged were less than 35 years and had a qualifying medical education. This could also explain their good level of knowledge and confidence in explaining the mode, duration of action, advantages, and disadvantages of contraceptive methods to adolescents and youth.

Even most providers had a positive attitude toward the use of modern contraceptive methods by adolescents and youth, some of them had social considerations that might influence their practices in providing FP services to adolescents and youth. Other studies have also revealed the presence of important contextual and social norms that lead to health providers negative attitudes toward the delivery of modern method contraceptive services to adolescents and youth (12, 13, 15–17, 21).

More than one-third (39%) of the providers felt that unmarried youth should not receive contraceptives because their culture does not support premarital sex. Nearly one out of four providers felt that it was immoral for adolescents and youth to use contraceptives. These results corroborate the findings of Nalwadda et al. in Uganda and Ahanonu in Nigeria, who reported that more than one-third of maternal and reproductive health providers said they would not provide contraceptives to unmarried, school-going, and childless people under age 18 because of social norms (12, 17). Health care providers may believe that they owe adolescents and unmarried youth a duty to protect them from sexual practice. However, this perception does not match reality, as evidenced by the high rates of sexual activity, sexually transmitted infections, and unwanted pregnancies and their implication for school dropout among this group. This negative attitude of health care providers is a major challenge to national efforts to ensure young people's right to sexual health, to protect their lives and their future. Positive attitudes among health care providers are a key element in improving contraceptive use among adolescents and youth. It is important that attitudes against contraceptive use by sexually active adolescents and youth be revised to reflect the realities of their sexuality. For example, sexual abstinence should be encouraged by providers for those adolescents and youth who are ready to practice it. For those who are sexually active and want to use contraceptives, access to contraceptive services should not be confronted with religious considerations, as it is better to prevent unwanted pregnancies and STIs than to face the consequences that may result. Only two in five providers had good practices regarding contraceptive use by adolescents and youth. This result is similar to those of other authors in Uganda (17, 28). This result could be explained by the persistence of the weight of socio-cultural factors such as religious beliefs on health care providers in delivering FP services.

Our results showed that being a midwife, having several years of professional experience, and residing in the Lower, Upper, middle, and Forest Guinea regions were simultaneously associated with positive attitudes and good provider practices toward modern contraceptive use by adolescents and youth. This result corroborates that of other authors in Nigeria (12) and in South Africa (29, 30) who argue that providers' region of residence, socio-professional category, and years of experience influence their attitudes and practices toward delivering FP services to adolescents. The simultaneous association of professional experience with positive attitudes and good practices could be explained by the acquisition of skills and confidence over the years in providing health services to adolescents and youth. The association of midwifery with positive attitudes and good practices regarding the use of modern contraceptive methods by adolescents and youth is explained by the frequent exposure of this group of providers to training on sexual and reproductive health issues, including among adolescents and youth.

The study also showed that the likelihood of having positive attitudes toward the use of modern contraceptive methods by adolescents and youth increased with provider age. This finding supports our initial observations of a positive relationship between increasing years of work experience and positive provider attitudes. It also corroborates the findings of Jonas et al. in South Africa, who found a positive association between the number of years of experience of health workers and their likelihood of providing maternal and child health services to adolescents (30). This result can be explained by the fact that the older the health worker, the more he or she is exposed to realities that influence his or her perceptions of adolescent contraception.

Our study also finds a positive association between gender, midwives' socio-professional category, and a positive provider attitude. Our results are consistent with those of Jonas et al. in South Africa, who found that years of experience as a nurse or midwife were associated with stronger intentions to provide FP services to adolescents (30). Furthermore, midwives are the ones most involved in the provision of contraceptive methods and therefore are exposed over time to training and work experience that improves their practices. Another explanation for the association between female gender and a positive provider attitude may be that women are more predisposed to understanding the needs of adolescents for birth control once they are sexually active, particularly when they are unmarried.

In addition, providers living in the countryside had better attitudes toward using modern contraceptive methods by adolescents and youth than providers in Conakry. The reason for this funding remained speculative. One possible reason is that health system actors put substantial efforts in improving health indicators in rural and remote areas, thereby neglecting health personnel capacity-building needs in the Capital. Further studies are needed to understand the underlying reasons for this finding.

Our results also showed that being a medical doctor and having several years of professional experience were significantly associated with provider practices in delivering modern methods to adolescents and youth. Medical doctors were also more likely to have good practices regarding the use of contraceptive methods by adolescents. This would be especially important if they are involved in providing reproductive health services in general. Our result would be explained by the fact that medical doctors are the ones who face the most serious consequences of adolescents' unmet need for contraception, such as complications from clandestine abortion. Paul et al. (30) in Uganda found that, beyond considerations for existing socio-cultural values of chastity, health personnel involved in delivering FP services had a better understanding of the vulnerability of girls and the consequences of adolescent pregnancy, and especially when they are unmarried.

### Limitations

Our study has some limitations that are inherent in any cross-sectional study. First, the cross-sectional nature of the study does not allow for a temporal relationship between the independent variables and the outcome of interest. Second, there is the possibility of social desirability bias that may lead to an overestimation of the level of good attitudes and practices in the delivery of FP services to adolescents and youth. However, the study has the advantage of using a sample that includes health personnel involved at several levels in the delivery of FP services to adolescents. It also has the advantage of having attempted to understand the factors that may influence the attitudes and practices of FP service providers for adolescents and youth in Guinea.

## Conclusion

Knowledge, attitudes, and practices of health care providers affect the delivery of FP services to adolescents and youth in Guinea. Most health care providers were confident in explaining their use, advantages, and disadvantages to adolescents and youth. However, a disparity exists between the level of Knowledge of health care providers and their attitudes and practices toward delivering FP services to adolescents and youth. Factors concurrently associated with positive attitudes and good practices of providers in this study included socio-professional category, number of years of experience in delivering contraceptive services, and region of residence. Future intervention programs should focus on training health care providers in youth- and adolescent-friendly reproductive health services and on popularizing regulations for adolescent contraception. This intervention should also include developing and implementing a continuing education program on the provision of contraceptive services for health providers who are nurses and midwives and have less than two years of experience in contraceptive provision. Additional efforts will also be needed to improve the attitudes and practices of health care providers toward the delivery of FP services in Guinea. Health care providers' attitudes should be based on the sexual realities of adolescents and youth. Further studies are needed to understand the underlying reasons for the health providers attitudes toward using modern contraceptive methods by adolescents and youth.

## Data availability statement

The raw data supporting the conclusions of this article will be made available by the authors, without undue reservation.

## Ethics statement

The studies involving human participants were reviewed and approved by National Committee of Ethics for Health Research in Guinea (No: 045/CNERS/19). Written informed consent to participate in this study was provided by the participants prior to participating in the study.

## Author contributions

SS, AD, DK, BSC, and SK designed the study and developed the study protocol. SS, FMG, and AD designed the analysis plan. SS, DK, and FMG, performed the data analyses, interpreted the results, and the manuscript was drafted by SS with critical inputs from DK, SK, BSC, and AD. All authors critically revised and approved the final manuscript.

## Funding

This research was part of a research scholarship provided by the International Union for the Scientific Study of Population (IUSSP) through the Grant No. OPP1179495 from the Bill & Melinda Gates Foundation. Additional funding to the lead author was provided by HRP. Alliance, part of The UNDP-UNFPA-UNICEF-WHO-World Bank Special Programme of Research, Development and Research Training in Human Reproduction (HRP), a cosponsored programme executed by the World Health Organization (WHO), through a PhD Scholarship granted by Institut Africain de Santé Publique (IASP/USTA) of the Université Saint Thomas D'Aquin, Burkina Faso. This article represents the views of the named authors only and does not represent the views of the World Health Organization.

## Conflict of interest

The authors declare that the research was conducted in the absence of any commercial or financial relationships that could be construed as a potential conflict of interest.

## Publisher's note

All claims expressed in this article are solely those of the authors and do not necessarily represent those of their affiliated organizations, or those of the publisher, the editors and the reviewers. Any product that may be evaluated in this article, or claim that may be made by its manufacturer, is not guaranteed or endorsed by the publisher.

## Abbreviations

AOR: Adjusted Odds Ratio
CI: Confidence Interval
DHS: Demographic and Health Survey
FP: Family Planning.
